# A longitudinal study of developmental quotients in early interventions for children with intellectual disability

**DOI:** 10.3389/fpsyt.2025.1639958

**Published:** 2025-10-07

**Authors:** Shaocheng Cai, Yu Wen, Yingjie Zhan, Liuliang Yuan

**Affiliations:** ^1^ Children’s Medical Center, Affiliated Hospital of Guangdong Medical University, Zhanjiang, China; ^2^ Department of Paediatrics, Zhanjiang Maternal and Child Health Hospital, Zhanjiang, China; ^3^ Department of Paediatrics, Southern Medical University Pingshan Hospital (Shenzhen Pingshan District People’s Hospital), Shenzhen, China; ^4^ Library, Guangdong Medical University, Zhanjiang, China

**Keywords:** intellectual disability, developmental quotients, Gesell Developmental Schedules, longitudinal study, early intervention

## Abstract

**Objective:**

This study aimed to examine the longitudinal relationships among different developmental quotients (DQs) in children with intellectual disability, focusing on the temporal dynamics of adaptive, language, and personal-social development over a two-year period, thereby informing more targeted early intervention strategies.

**Methods:**

We retrospectively analyzed data from 94 children (72 males, 22 females, aged 2–5 years) diagnosed with intellectual disability who had continuously received behavioral interventions for neurodevelopmental disorders at our hospital over a two-year period. The dataset included demographic information and DQs for adaptive behavior, language ability, and personal-social skills, which were assessed using the Chinese version of Gesell Developmental Scale. Cross-lagged panel modeling was conducted using Mplus 8.3 to examine the correlations among DQ domains at the same time point, their stability across time, and cross-domain predictive relationships.

**Results:**

The reciprocal associations model, which incorporated cross-lagged paths to the stability model, demonstrated good fit. At all time points, adaptive, language, and personal-social DQs were moderately to strongly correlated. Each domain showed high temporal stability, as indicated by significant autoregressive paths. Adaptive DQ at Time 1 and Time 2 significantly predicted language and personal-social DQs at subsequent time points (β = 0.272–0.337, p < 0.01). Additionally, language DQ at Time 1 predicted personal-social DQ at Time 2. In contrast, neither language nor personal-social DQ predicted future adaptive DQ.

**Conclusion:**

Adaptive functioning is prospectively associated with later development of language and personal-social skills in children with intellectual disability. Early interventions that prioritize adaptive skill development may yield broader benefits across other developmental domains. Further research is warranted to develop and evaluate intervention models that strategically leverage these directional relationships to optimize long-term outcomes.

## 1 Introduction

Intellectual disability (ID) is a prevalent childhood neurodevelopmental disorder, with an estimated prevalence of 1–3% (1, 2). It’s characterized by onset during the developmental period and significant impairments in both cognitive and adaptive functioning (3). Although ID encompasses a heterogeneous spectrum of clinical presentations, growing evidence indicates that early behavioral interventions can lead to significant and lasting developmental gains in infants diagnosed with ID (4–6). Owing to its high prevalence, substantial long-term socioeconomic burden, and enduring impact on individual functioning, early intervention for children with ID is critically important (7).

To facilitate children with ID in accessing targeted diagnostic and intervention services, researchers have extensively investigated diagnostic instruments, intervention approaches, and methods for evaluating intervention outcomes over the past decades. Widely used tools for assessing intellectual functioning include the Wechsler Intelligence Scale and the Stanford–Binet Intelligence Scale. Cognitive abilities are commonly evaluated using the Mullen Scales of Early Learning, adaptive functioning is measured with the Vineland Adaptive Behavior Scales, and overall developmental progress is assessed via the Bayley Scales of Infant Development. In parallel, a range of intervention strategies has been developed to target multiple developmental domains, including cognition, language, motor skills, socio-emotional functioning, and adaptive behavior (8–12).

Longitudinal studies of neurodevelopmental disorders, particularly those adopting developmental cascade models, have shown that progress or delays in one domain—such as language, cognition, social functioning, or adaptive behavior—not only shape outcomes within that specific domain but also exert reciprocal influences on other domains over time (13–16). In children with ID, the development of cognition, language, motor skills, socio-emotional functioning, and adaptive behavior may be a dynamic and interrelated process, various intervention strategies may exert their effects within these dynamic structural relationships.

### 1.1 The developmental cascades framework applied to the study of ID

Current longitudinal research on neurodevelopmental disorders has predominantly focused on autism, particularly on studies that apply the developmental cascade framework to examine associations among skills, behaviors, and traits in children with neurodevelopmental disorders. For example, Baribeau et al. investigated longitudinal associations between anxiety symptoms and insistence on sameness in autistic children using a developmental cascade model (17). Bennett et al. explored the bidirectional links between language ability and social functioning (18), while Oosting et al. examined reciprocal associations between language development and social functioning in preverbal autistic children using a developmental cascade model (13). Bottema-Beutel et al. analyzed developmental associations between joint engagement and vocabulary growth through cross-lagged panel analysis (19). Wei et al. reported bidirectional relationships between infants’ gross motor development and physical growth (14), and Li et al. identified associations between early language and motor abilities and later autistic traits using random intercept cross-lagged panel models (20). In addition, Ravi et al. employed a longitudinal approach to examine the relationship between social communication skills and language development in infants who were subsequently diagnosed with autism (21).

Compared with autism research, applications of the developmental cascade framework to the study of ID remain relatively limited, although several notable studies illustrate its potential. In 2019, Schuengel et al. conducted a comprehensive systematic review of longitudinal studies on the early development of children with ID (22), revealing that despite its critical importance, this area remains relatively underexplored. Wang et al. conducted a two-year longitudinal study and found that working memory predicts receptive vocabulary in children with ID (23). van der Schuit et al. investigated the impact of cognitive factors on language development in children with ID (24). Wang, QQ et al. investigated the longitudinal relationship between the home literacy environment and reading ability in children with ID (25). Hofmann, V. and Müller, CM studied the relationship between language skills and social contact among students with ID in special needs schools (26).

Conducting longitudinal studies on children with ID involves substantial methodological and practical challenges (22). First, limited communicative and cognitive abilities often hinder the collection of reliable self-reports, and even when obtained, such reports are frequently viewed as lacking validity by clinicians and researchers. Second, recruiting sufficiently large and representative samples of young children and their families is particularly demanding, yet essential for constructing statistically robust developmental models and testing hypotheses about underlying mechanisms. Such efforts typically require extensive resources and cross-institutional collaboration, often spanning multiple research groups and countries. A further challenge lies in the early identification of ID, as intellectual and adaptive functioning in young children show considerable variability across both time and individuals, rendering early and definitive diagnoses highly complex. Due to the instability of developmental profiles and the potential uncertainty of confirming intellectual impairment, in international clinical and research practice, children under 5 years of age with intellectual disabilities are often classified as having Global Developmental Delay. Finally, longitudinal studies in this population are especially vulnerable to high attrition rates, which further undermine statistical power and limit the generalizability of findings.

### 1.2 Research on Developmental quotients in ID

DQs are widely used in China as auxiliary indices for diagnosing and evaluating neurodevelopmental disorders. Prior research indicates that children with ID often show delays or plateaus across multiple DQ domains, including adaptive behavior (coordination, imitation, discriminative performance and perception), language behavior (use of vocabulary, language comprehension and conversational skills), and personal-social behavior (social habits, reactions to persons, autonomy and acquired information (27, 28). DQs serve as comprehensive indices for evaluating the maturational level across multiple developmental domains in children (29). Deficiencies across DQs in children with ID can exert profound and long-lasting effects on their daily functioning, learning capacity, and overall quality of life (30). Targeted interventions aimed at improving DQs hold significant promise in optimizing developmental trajectories and improving long-term functional outcomes in children with intellectual disability (31).

In recent years, Chinese researchers have utilized the Gesell Developmental Schedules in over 2,000 published studies as standardized instruments to assess DQs across a range of neurodevelopmental disorders (32, 33). These studies have also examined the antecedents and long-term outcomes associated with DQs (34–36), demonstrating that various intervention strategies can significantly enhance developmental outcomes. In addition, some studies have explored the interrelationships among different DQs. For instance, Tao C found that delayed language development was frequently accompanied by abnormalities in motor skills, adaptive behavior, and personal-social functioning (37). Zheng X.F. et al. (2016) revealed statistically significant positive correlations between the DQ for language and the DQs for both adaptive and social domains (27). In 2019, Li P et al. found that the DQs were positively correlated, suggesting that different developmental domains may interact and complement each other (24, 38). However, much of the existing literature places disproportionate emphasis on language development, frequently positing it as the central factor shaping other DQs. This focus may risk oversimplifying the complex interdependencies that exist among DQs. In particular, no longitudinal study has systematically examined the directional interplay among DQs in children with ID, and no longitudinal study has systematically examined how intervention strategies leverage these interdependent mechanisms to enhance developmental outcomes in children with ID. Addressing this gap is crucial for optimizing early intervention strategies and advancing our understanding of the complex developmental processes in this population.

### 1.3 Overview of the present study

The developmental cascade model provides a conceptual framework for examining how progress in one aspect of a developing system can trigger widespread and enduring effects across other developmental domains. Despite its utility, longitudinal research on DQs in early interventions for children with ID remains limited. To address this gap, we selected 94 children from a larger cohort who had continuously received behavioral interventions for neurodevelopmental disorders at Zhanjiang Maternal and Child Health Hospital between July 2020 and December 2022. Guided by the developmental cascade framework, we conducted a three-wave longitudinal study to investigate the stability of, and cross-domain relationships among, adaptive, language, and personal-social DQs in children with ID.

The specific objectives were: (1) to assess the stability of each DQ domain over time (i.e., whether adaptive, language, and social DQs at Time 1 predict themselves at Time 2 and Time 3), and (2) to explore the cross-lagged relationships among adaptive area, language area, and social area over time (i.e., whether an earlier domain predicts changes in a different domain over time). We hypothesized that all areas would exhibit significant stability and concurrent inter-correlations across time.

Based on the developmental cascade model, we first examined the following three relationships: (1) early adaptive DQ significantly predicts later language and personal-social DQs; (2) early language DQ predicts later adaptive and personal-social DQs; and (3) early personal-social DQ predicts later adaptive and language DQs. Guided by Gesell’s theory, which posits that adaptive behavior serves as a precursor to later intelligence (39), we hypothesized that early adaptive DQ exerts cascading effects on both language and personal-social DQs. Building on Li et al.’s hypothesis (38), which suggests that language may interact with and complement other developmental domains, we further hypothesized that early language DQ similarly has cascading effects on both adaptive and personal-social DQs.

**Figure 1:**
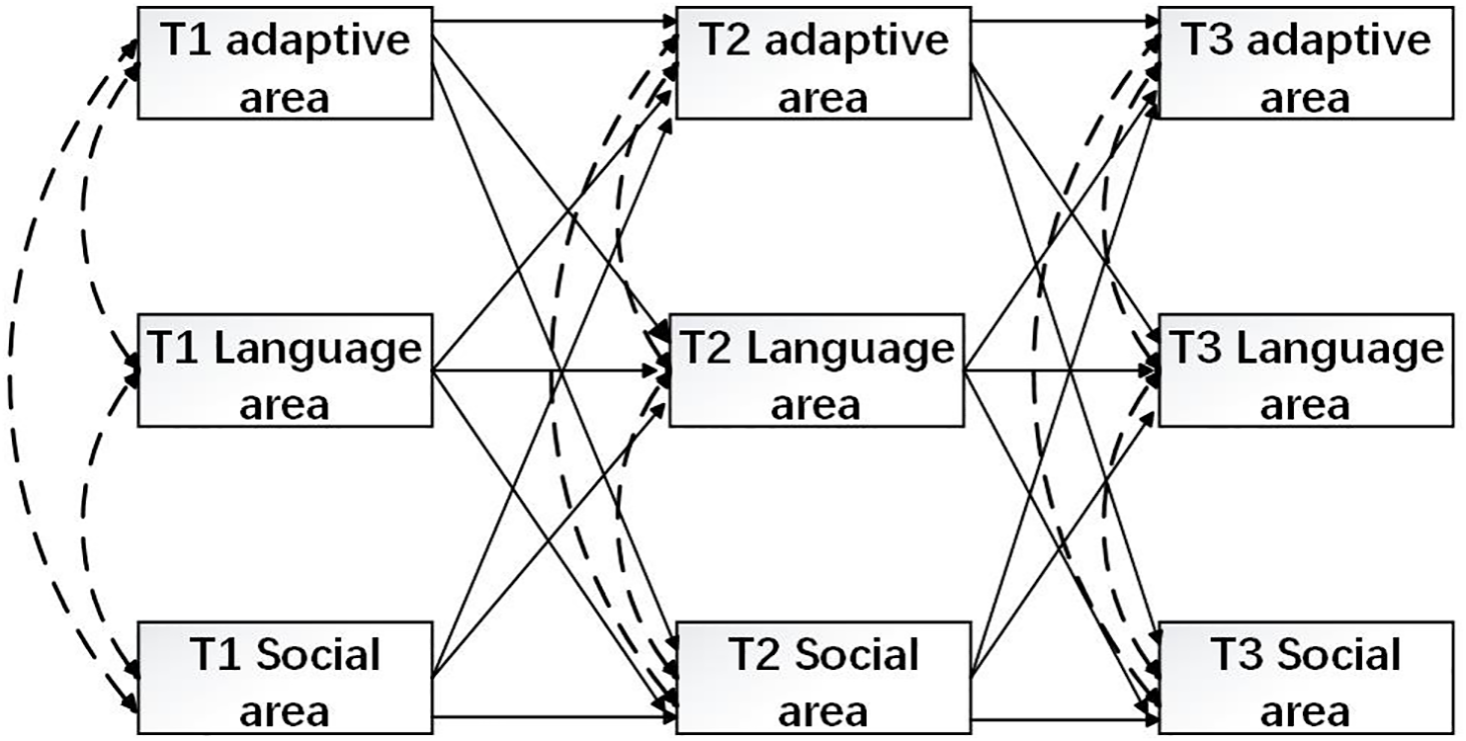
Conceptual framework.

These hypotheses are illustrated in the conceptual model (**Figure 1**).

## 2 Materials and methods

### 2.1 Participants

The study was approved by the Ethics Committee of Zhanjiang Maternal and Child Health Hospital. We retrospectively selected 94 children (72 boys, 22 girls) aged 2–5 years who had been diagnosed with ID and treated at Zhanjiang Maternal and Child Health Hospital between July 2020 and December 2022.

Inclusion criteria. Participants met the following criteria: (1)A formal diagnosis of intellectual disability in accordance with the Diagnostic and Statistical Manual of Mental Disorders, Fifth Edition (DSM-V). (2)A chronological age between 2 and 5 years. (3)Developmental delay evidenced by a Developmental Quotient (DQ) of ≤ 75 in at least two domains—adaptive behavior, gross motor skills, fine motor skills, language, or social interaction—assessed using the Gesell Developmental Scale. (4)Provision of written in-formed consent by a parent or legal guardian.

Exclusion criteria. Exclusion criteria were as follows: (1)Presence of genetic syndromes, medically confirmed autism spectrum disorders, or motor impairments severe enough to preclude reliable developmental assessment. (2)Absence of documented in-formed consent. (3)Intellectual disability attributable to known etiological factors such as traumatic brain injury, intracranial infection, or toxic environmental exposure.

Among the 94 enrolled children, the completer group (n = 53) consisted of those who completed assessments at all three time points (initially between 15 and 55 months of age, approximately one year later, and a further year later), whereas the non-completer group (n = 41) was assessed only at Time 1 and Time 2. Missing data at Time 3 (from the non-completer) were handled using Full Information Maximum Likelihood (FIML) estimation in Mplus 8.3, which is recommended for yielding accurate estimates with incomplete longitudinal data (25, 26). Little’s MCAR test confirmed that the missing Time-3 data were missing completely at random (p > 0.05), justifying the use of FIML. Key demographic characteristics of the sample are summarized below (**Table 1**): Age (months): Time1 mean = 40 (SD 10); Time2 mean = 52 (SD 11); Time3 mean = 64 (SD 10). Gender: 72 males (76.5%), 22 females (23.5%). Parental Education: High school or less 47 (50.0%); Associate degree (2-year) 14 (14.9%); Bachelor’s degree 32 (34.0%); Postgraduate 1 (1.1%). Comparisons between the completer and non-completer groups on three key variables—age, sex, and baseline DQ—were conducted. Independent-samples t-tests revealed no significant difference in age between the two groups (t = 0.815, p = 0.418 > 0.05), and no significant difference in baseline DQ (t = 1.55, p = 0.124 > 0.05). A chi-square test further indicated that the distribution of sex did not differ significantly between the groups (χ² = 0.026, p = 0.872).

**Table 1:** Participant sociodemographic information.

Child characteristics	Mean/Number	SD/Percentage
Age (months)	Mean	SD
TIME1	40	10
TIME2	52	11
TIME3	64	10
Gender	N	%
Male	72	76.5
Female	22	23.5
Parents Educational level	N	%
High school/lesser	47	50
Associates or 2-year degree	14	14.9
Bachelor	32	34
Postgraduate	1	1.1

### 2.2 Intervention program

Pre-intervention assessment. Before initiating rehabilitation, all children underwent a standardized baseline evaluation. The assessment battery included hearing and vision screening, developmental testing with Chinese norms (Gesell Developmental Scale), adaptive behavior assessment (ABAS-II), language evaluation (S-S), and functional assessments of daily living from infancy through middle childhood. Diagnosis of intellectual disability was made in accordance with the DSM-V criteria. Autism spectrum disorder screening was also conducted using the parent-report version of the Autism Behavior Checklist (ABC). Based on these assessments, along with clinical observations and multidisciplinary evaluations, physicians formulated an individualized education plan (IEP) tailored to each child’s developmental profile. Rehabilitation interventions and home-based guidance were subsequently initiated after obtaining written informed consent from parents or legal guardians.

Intervention model. Interventions were implemented using the Early Start Denver Model (ESDM), a validated and evidence-based framework specifically designed for children aged 12–72 months with developmental delays. The ESDM employs a play-based, child-centered approach that emphasizes responsiveness to children’s communicative behaviors (e.g., eye gaze, gestures, vocalizations) and provides immediate positive reinforcement to enhance social engagement. Intervention strategies were embedded within naturalistic routines and targeted multiple developmental domains—including language, cognition, and social skills—to promote broad improvements in communication, adaptive functioning, and learning readiness.

Implementation. Children participated in intensive training, with a minimum of 20 hours per week (≥5 hours per day) combining clinic-based and home-based sessions. All interventions were delivered by licensed pediatric rehabilitation specialists with at least three years of clinical experience and formal certification in ESDM implementation. In-clinic training was conducted five days per week for 1–2 hours per session. Activities were tailored to individual developmental levels, beginning with one-on-one instruction and progressively transitioning to small-group interactions (2–4 children) and simulated classroom settings (6–8 children). Play-based methods and interactive scenarios were consistently employed to foster positive social relationships, self-initiation, and active participation. The intervention addressed language, cognitive, social, and motor domains, with a strong emphasis on skill generalization in natural contexts. Positive affective engagement and reinforcement strategies were systematically applied to strengthen social motivation and sustain learning interest.

Parent training and home involvement. Parental involvement was an integral component of the intervention. Parent-coaching sessions were conducted five days per week for 1–2 hours, with parents actively engaging alongside their children and receiving guidance on incorporating ESDM strategies into everyday routines such as mealtimes and play. Additionally, individualized parent guidance sessions were provided twice weekly (1 hour each). During these sessions, therapists offered feedback, addressed implementation challenges, and guided parents in extending intervention strategies to family activities (e.g., parent–child games, household routines). Digital platforms were also utilized to share video-based instructional content, enabling timely correction of implementation errors and fostering continuous parent–therapist communication. This combination of professional intervention and parental involvement ensured fidelity of implementation and supported the maintenance and generalization of intervention effects in the home environment.

### 2.3 Measures

The Chinese version of Gesell Developmental Scale, which has demonstrated strong reliability, validity, and internal consistency in assessing neurodevelopment in children (40, 41), was used as a standardized instrument to assess children’s DQs in three major domains: adaptive area, language area, and Personal-social area.

The Gesell Developmental Scale assesses children’s developmental levels by using the behavioral patterns of typically developing children as a reference standard for identifying and evaluating observed behaviors. Developmental levels are quantified in terms of developmental age and DQ. The DQ was calculated by dividing the developmental age by the chronological age and multiplying the result by 100. According to the criteria established by the Gesell Developmental Scale, Children’s developmental levels were classified as normal (DQ ≥ 85), borderline (75 ≤ DQ < 85), or abnormal (DQ < 75). A DQ score of less than 75 in any functional domain was indicative of abnormal development (42).

In this study, all assessments were conducted by a single rehabilitation physician in accordance with the standardized procedures of the Gesell Developmental Scale. Evaluations were performed in a quiet, private, and well-lit room. Family members were permitted to accompany the children during the assessments to facilitate optimal performance.

### 2.4 Data analysis

To investigate the bidirectional associations among adaptive behavior, language behavior, and personal-social behavior, we employed a cross-lagged panel design and conducted within-domain path analyses across three waves (Time 1, Time 2, and Time 3). Model fit was evaluated using multiple fit indices, with the following criteria: a non-significant chi-square test (p > 0.05), Comparative Fit Index (CFI ≥ 0.95), Tucker–Lewis Index (TLI ≥ 0.95), Root Mean Square Error of Approximation (RMSEA ≤ 0.06), and Standardized Root Mean Square Residual (SRMR ≤ 0.08) (43). To assess whether the missing data at Time 3 were missing completely at random, Little’s MCAR test was conducted using SPSS. Missing values were handled using Full Information Maximum Likelihood (FIML) estimation in Mplus 8.3.

**Figure 2:**
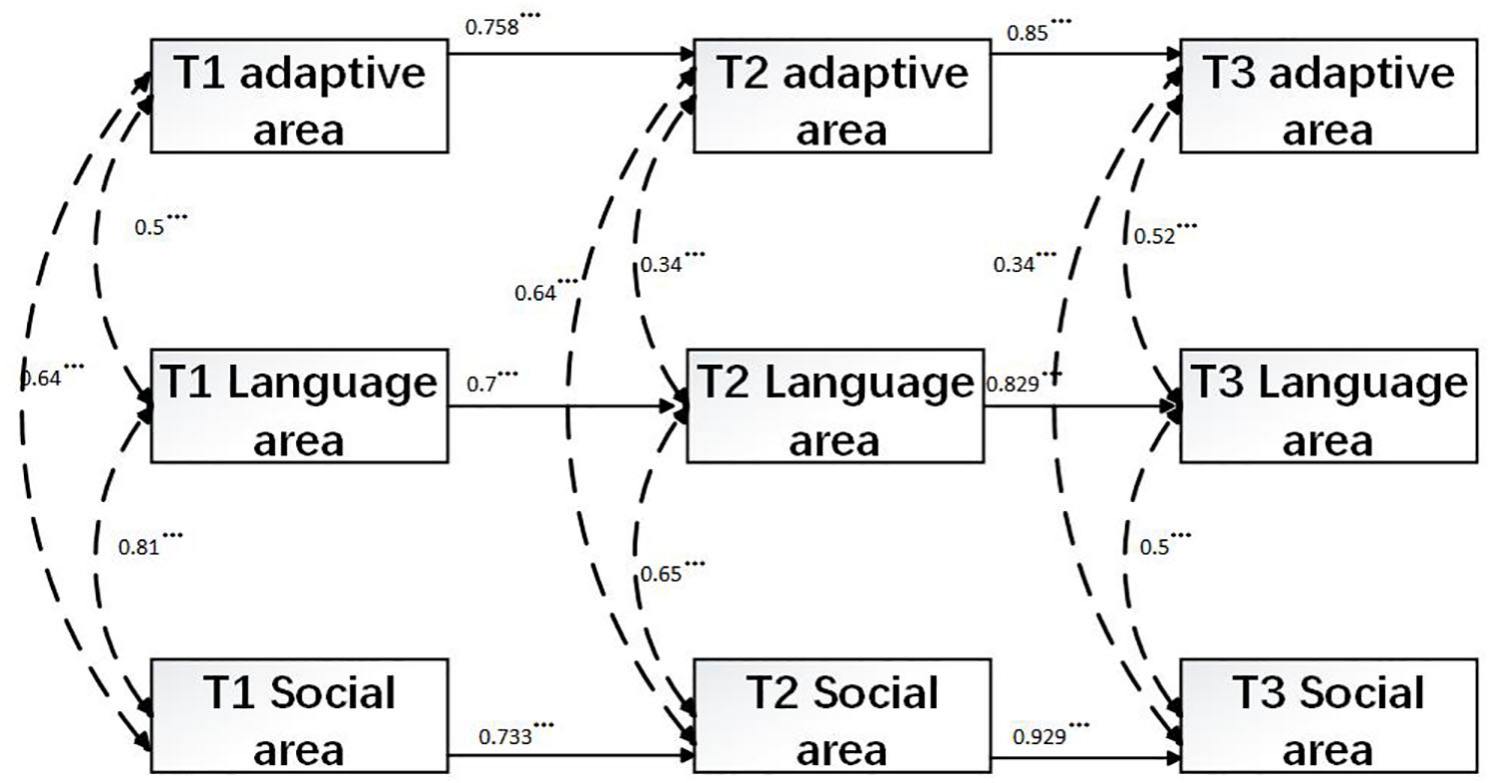
Stability model. Parental education level was controlled. The two-headed arrows represent concurrent correlations. The numbers on these arrows indicate the Pearson’s correlation coefficient, which measures the linear relationship between two variables. The values on the single-headed arrows represent standardized coefficients. ***p <.001.

We first tested a baseline model that included stability paths for all constructs across time points (i.e., from Time 1 to Time 2 and from Time 2 to Time 3), as well as the mediating effects within the monitoring model. Additionally, concurrent correlations among adaptive behavior, language behavior, and personal-social behavior within each assessment period were incorporated (See **Figure 2** stability model).

To further investigate the directional dynamics among the constructs, we conducted a cross-lagged panel analysis. In this extended model, directional paths were specified across time points among adaptive behavior, language behavior, and personal-social behavior. Specifically, Time 2 adaptive behavior was regressed on Time 1 adaptive behavior, language behavior, and personal-social behavior. Similarly, Time 2 language behavior and Time 2 personal-social behavior were each regressed on all three constructs at Time 1. These predictive relationships were then examined again from Time 2 to Time 3, maintaining the same structural framework. Concurrent correlations among adaptive behavior, language behavior, and personal-social behavior within timepoint were also included (See **Figure 1**).

## 3 Results

### 3.1 Descriptive analyses

We conducted descriptive statistical analyses and paired-sample t-tests using Mplus 8.3, applying full information maximum likelihood estimation to address missing data. Observed raw scores on the adaptive, language, and personal-social DQs subdomains improved significantly over time for the sample on average (**Table 2**). For adaptive DQ, the mean score increased significantly from 57.91 (SD = 1.71) at T1 to 60.37 (SD = 1.75) at T2, t = 3.11, p < 0.01. From T2 to T3, scores further improved from 60.37 (SD = 1.75) to 60.75 (SD = 2.48), t = 3.83, p < 0.01. Language DQ showed significant growth, with mean scores rising from 42.44 (SD = 1.81) at T1 to 44.60 (SD = 1.79) at T2, t = 4.08, p <.01. A further significant increase was observed between T2 and T3, from 44.60 (SD = 1.79) to 48.44 (SD = 1.99), t = 3.97, p < 0.01. Similarly, personal-social DQ demonstrated significant gains over time. From T1 to T2, scores increased from 51.26 (SD = 1.44) to 52.39 (SD = 1.43), t = 3.66, p <0.01. From T2 to T3, further improvements were observed, with mean scores rising from 52.39 (SD = 1.43) to 53.81 (SD = 1.65), t = 3.84, p < 0.01. Together, these findings suggest sustained developmental progress over time, likely attributable to the interventions administered during the assessment period.

**Table 2:** Changes in adaptive, language, and personal-social behaviors across three time points.

Construct	T1 mean (SD)	T1-T2(t)	T2 mean (SD)	T2-T3(t)	T3 mean (SD)
adaptive behavior	57.91(1.71)	3.113**	60.37(1.75)	3.834**	60.75(2.48)
language behavior	42.44(1.81)	4.083**	44.6(1.79)	3.969**	48.44(1.99)
personal-social behavior	51.26(1.44)	3.663**	52.39(1.43)	3.842**	53.81(1.65)

Values are presented as means (standard deviations). Paired-sample t-tests were used to examine changes between consecutive time points.

**p <.01.

### 3.2 Stability model

The stability model incorporated autoregressive pathways across two consecutive time intervals— specifically, from Time 1 to Time 2 and from Time 2 to Time 3—for adaptive behavior, language behavior, and personal-social DQs. In addition to capturing these temporal stability paths, the model accounted for the mediating mechanisms embedded within the monitoring framework, as well as the concurrent correlations among the three constructs at each time point (see **Figure 2**). The mediating effects in the model were found to be statistically significant (see **Table 3**). The autoregressive pathways indicated significant temporal stability for all three constructs for all three constructs, with significant stability over time. Concurrent correlations between the constructs at each time point were also significant, ranging from moderate to strong (0.34 to 0.81). Despite the robust longitudinal and concurrent relationships observed, the model fit indices indicated that the model did not provide an adequate fit to the data (χ²(21) = 43.975, p < 0.01; CFI = 0.941; TLI = 0.907; RMSEA = 0.139; SRMR = 0.183).

**Table 3:** Mediating effects of stability model .

Path	Standardized path coefficient	SE	Two-tailed	95% confidence interval	
			P-value	Lower	Upper
Time1 adaptive→Time2 adaptive→Time3 adaptive	0.644	0.085	0.000	0.493	0.774
Time1 language→Time2 language→Time3 language	0.580	0.070	0.000	0.447	0.677
Time1 social→Time2 social→Time3 social	0.681	0.073	0.000	0.551	0.793

### 3.3 Reciprocal associations model

**Figure 3:**
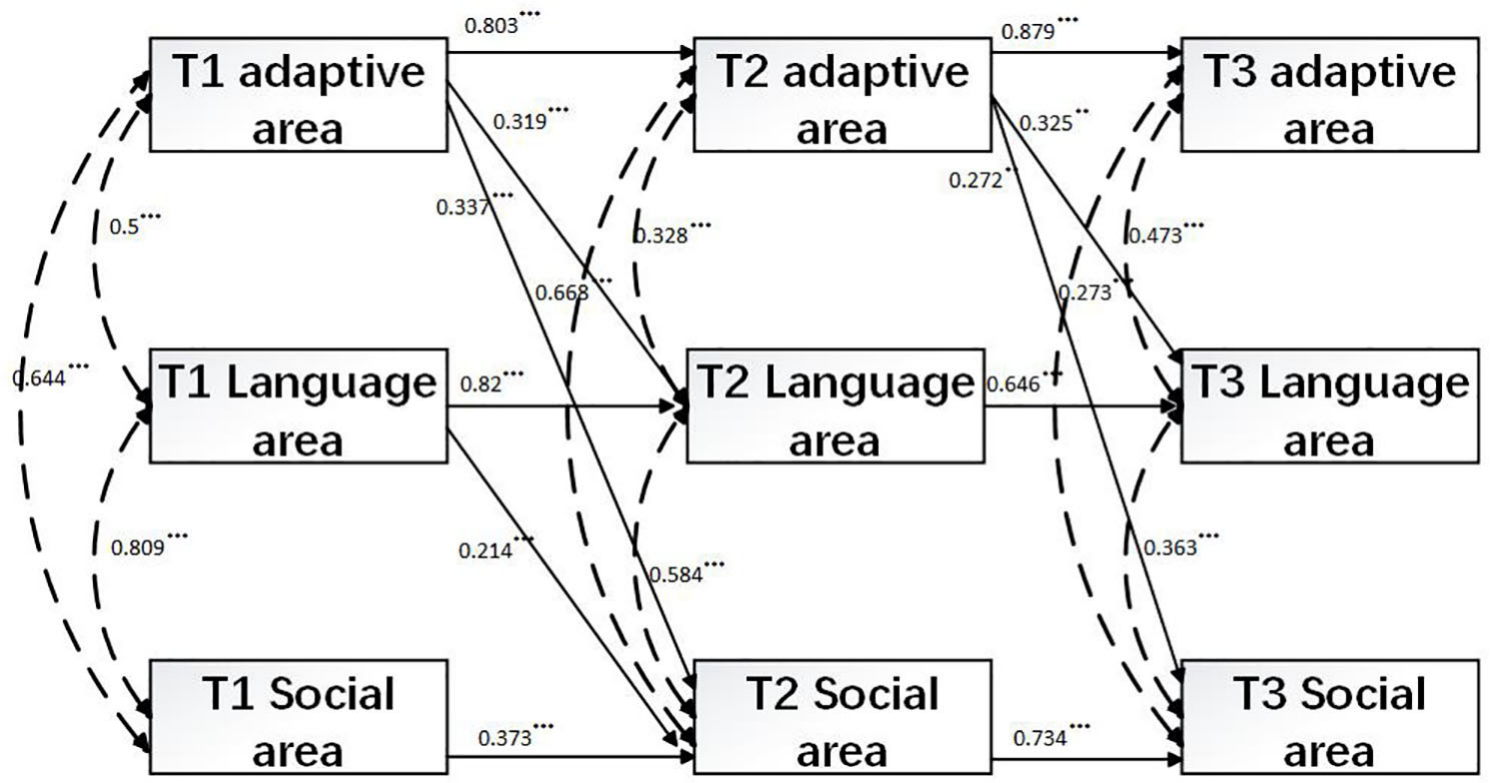
Reciprocal associations model. Parental education level was controlled. The two-headed arrows represent concurrent correlations. The numbers on these arrows indicate the Pearson’s correlation coefficient, which measures the linear relationship between two variables. The values on the single-headed arrows represent standardized coefficients, while the dashed lines of single-headed arrows indicate nonsignificant paths. ***p <.001, **p <.01.

The reciprocal associations model introduced cross-lagged paths to the stability model described above. Chi-square difference testing confirmed that the addition of these paths significantly enhanced model fit compared to the stability model (Δχ²(21) = 43.97, p = 0.002) (44, 45). With the inclusion of 18 cross-lagged paths, model fit substantially improved, with all five fit indices indicating an acceptable fit (χ²(9) = 10.29, p = 0.41; CFI = 0.998; TLI = 0.997; RMSEA = 0.02; SRMR = 0.049). Moreover, the autoregressive paths for adaptive DQ, language DQ, and personal-social DQ were all statistically significant, indicating strong stability over time (see **Table 4**). The concurrent correlations among adaptive DQ, language DQ, and personal-social DQ at each time point were also statistically significant, ranging from 0.34 to 0.81 (see **Figure 3**). Furthermore, Time1 adaptive DQ significantly predicted Time 2 language DQ and personal-social DQ, while Time 2 adaptive DQ significantly predicted Time 3 language DQ and personal-social DQ. Additionally, Time 1 language DQ significantly predicted Time 2 personal-social DQ.

**Table 4:** Mediating effects of reciprocal associations model.

Path	Standardized path coefficient	SE	Two-tailed	95% confidence interval	
			P-value	Lower	Upper
Time1 adaptive→Time2 adaptive→Time3 adaptive	0.706	0.12	0.000	0.508	0.904
Time1 language→Time2 language→Time3 language	0.530	0.146	0.000	0.29	0.769
Time1 social→Time2 social→Time3 social	0.274	0.1	0.006	0.109	0.439

However, contrary to expectations, Time 1 language DQ did not significantly predict Time 2 adaptive DQ, nor did Time 2 language DQ significantly predict Time 3 adaptive DQ or personal-social DQ. Similarly, Time 1 personal-social DQ did not significantly predict Time 2 adaptive DQ or language DQ, and Time 2 personal-social DQ did not significantly predict Time 3 adaptive DQ or language DQ.

## 4 Discussion

Guided by a developmental cascade framework, the present study examined both the within-domain longitudinal stability and the cross-lagged associations among adaptive behavior, language behavior, and personal-social behavior at three time points in young children with ID employing the cross-lagged panel analysis. The analyses not only confirmed the interrelations among the three developmental domains at each time point and demonstrated robust within-domain stability across time, but also revealed that early adaptive behavior serves as a positive prognostic indicator for subsequent language and personal-social development.

### 4.1 Application of the developmental cascades framework to research on ID

The present findings validate the utility of the developmental cascades framework in examining the interrelationships among skills, behaviors, and traits in children with ID. Previous applications of this framework in autism research have primarily relied on internationally recognized assessment tools such as the Vineland Adaptive Behavior Scales, Bayley-III, and Mullen Scales of Early Learning (20, 46). In contrast, the current study extends the application of the developmental cascade framework to DQs assessed using the Chinese version of the Gesell Developmental Scale, thereby broadening its relevance to research on neurodevelopmental disorders.

Prior research has shown that the developmental cascades framework captures the sequential, multilevel, and cross-domain nature of human development, making it particularly suitable for elucidating how interconnected systems exert far-reaching effects in both typical and atypical development (47, 48). Importantly, the Chinese version of the Gesell also evaluates gross motor and fine motor behaviors, providing opportunities for future studies to apply the developmental cascades framework to examine cascading relationships across all DQ domains—including gross motor, fine motor, language, adaptive, and social domains—in children with ID. Furthermore, future research should incorporate widely used international assessment tools to measure the skills, behaviors, and traits of children with ID and directly compare these findings with those derived from the Gesell. Such comparisons will be critical for testing the consistency and generalizability of cascade effects across different measurement frameworks.

### 4.2 Relationships and within-domain stability

The current study revealed significant correlations among adaptive behavior, language behavior, and personal-social behavior across all three assessment time points (Time 1, Time 2, and Time 3). This finding is consistent with prior research, confirming that the development of these three domains is interrelated and coordinated. Descriptive statistical analyses and analyses of variance further demonstrated that the mean values of adaptive behavior, language behavior, and personal-social behavior showed a steady upward trajectory from Time 1 through Time 3.

Longitudinal analyses indicated robust within-domain associations across all three dimensions. Moreover, adaptive behavior, language behavior, and personal-social behavior at Time 2 mediated the relationships between their corresponding measures at Time 1 and Time 3. From the perspective of autoregressive pathways and mediation effects, these results suggest that with increased intervention exposure, the development of the three domains improves in a progressive manner. Importantly, developmental status at adjacent time points not only predicted subsequent growth within the same domain but also demonstrated that earlier development (Time 1) exerted significant indirect effects on later development (Time 3) through mediating processes occurring at Time 2.

These findings indicate that although children with ID typically exhibit relatively slow growth in adaptive, language, and personal-social DQs, effective interventions—such as the Early Start Denver Model, play-based training programs, and caregiver coaching—can facilitate steady and meaningful developmental gains in these domains.

### 4.3 Reciprocal associations among DQs

Compared to the stability model, the cross-lagged model demonstrated significantly improved fit indices, indicating an excellent overall model fit. This suggests that incorporating reciprocal associations among adaptive behavior, language behavior, and personal-social behavior is both statistically justified and theoretically necessary.

The current study further revealed that adaptive behavior predicted subsequent changes in both language and personal-social behaviors over time. Specifically, adaptive behavior at Time 1 and Time 2 significantly predicted language and personal-social behaviors at Time 2 and Time 3, respectively. This finding suggests that the acquisition of new adaptive skills provides children with intellectual disability greater access to their environments and expanded opportunities for learning, thereby laying the foundation for progressively more sustained and sophisticated interactions with objects and people—interactions that, in turn, support the development of language and social competence. These findings are consistent with the theoretical perspective of Gesell (29), who posited that adaptive behavior serves as the forerunner of later intelligence, enabling children to apply prior experiences to the solution of novel problems. This underscores the critical role of early interventions that specifically target adaptive behavior in children with ID, as improvements in this domain may yield cascading developmental benefits across multiple functional areas. In other words, higher levels of adaptive functioning may facilitate more advanced language and social development.

In addition, language behavior at Time 1 significantly predicted personal-social behavior at Time 2.

However, language behavior at Time 2 did not significantly predict personal-social behavior at Time 3. These findings challenge the widely held assumption that language development acts as the primary driver of broader developmental progress in children with ID. Instead, they suggest that the cross-domain influence of language may be stage-dependent: once a certain threshold of language growth is reached, further gains may no longer directly translate into higher levels of communicative or social competence.

Finally, neither language behavior nor personal-social behavior at Time 1 or Time 2 significantly predicted adaptive behavior at Time 2 or Time 3. One possible explanation is that, among young children with intellectual disability in China, adaptive functioning may rely less on language and social skills at early developmental stages. Adaptive behavior is rooted in children’s interactions with their physical and social environments, but in younger children with intellectual disability, limited language and social abilities may be insufficient to support the advancement of adaptive skills through communicative exchanges with others.

### 4.4 Clinical and educational implications

The findings of this study have important implications for both clinical practice and educational interventions for children with ID. First, the observed predictive role of early adaptive behavior in later developmental outcomes suggests that early intervention programs should prioritize enhancing adaptive skills. While some researchers have thereby speculated that language behavior plays a causal role in the development or progression of DQs, these longitudinal findings show that adaptive behavior is a stronger predictor of future DQs than language behavior is of future DQs. Targeting these skills could provide a strong foundation for improving language and personal-social behaviors, ultimately leading to more holistic developmental gains.

Given that language behavior did not significantly predict subsequent adaptive behavior or personal-social behavior at Time 3, it is imperative for clinicians and educators to adopt a more individualized and developmentally informed approach to early intervention. For preschool-aged children— particularly those who continue to exhibit marked language delays despite receiving various forms of language-focused interventions—shifting the focus toward enhancing adaptive functioning or other foundational developmental domains may lead to more favorable long-term outcomes. This perspective further underscores the critical importance of implementing multidisciplinary intervention frameworks that holistically address the multifaceted nature of child development, rather than placing disproportionate emphasis on language acquisition alone.

Moreover, the lack of significant predictive relationships between personal-social behavior and the other domains further highlights the complexity of social development in children with ID. Interventions aimed at improving social behaviors may need to be more nuanced and context-dependent, addressing both intrinsic developmental factors (e.g., emotional regulation) and extrinsic influences (e.g., peer interactions, family dynamics). Social skills training that incorporates family and community involvement might prove to be an effective approach.

### 4.5 Limitations and future directions

Despite its strengths, this study has several limitations that should be addressed in future research. First, the study relied on a relatively small sample, drawn from a single hospital and predominantly male (76%). In addition, all assessments were conducted by a single rehabilitation physician, which may introduce observer bias. These characteristics limit the generalizability of the findings. Future studies should aim to replicate these results with a larger and more diverse sample to confirm the robustness of the observed relationships.

Additionally, while the use of the Gesell Developmental Scale provides a reliable and widely accepted measure of DQs, it is important to acknowledge that age-normed developmental measures may not fully capture the nuanced developmental trajectories of children with ID. Future research could consider incorporating other internationally recognized assessment tools or observational measures to provide a more comprehensive and comparable picture of developmental progress.

Furthermore, the present study did not stratify children with ID based on their level of intellectual functioning. Given the heterogeneity of cognitive profiles within this population (49), failing to account for different levels of intellectual impairment may obscure potential variations in developmental cascades across subgroups. Future research should therefore consider subgroup analyses based on the severity of intellectual disability, which may reveal distinct patterns of interrelationships among DQS.

## 5 Conclusions

This study highlights the importance of the developmental cascades framework in understanding the interrelationships between adaptive, language, and personal-social behaviors in children with ID. The findings indicate that early adaptive behavior is a strong predictor of later developmental progress in both language and social domains, emphasizing the need for interventions that prioritize adaptive skills. These results challenge the assumption that language behavior is the primary driver of broader developmental progress, suggesting that the impact of language on social development may be stage-dependent. In clinical practice, enhancing adaptive functioning may present a more effective strategy for fostering long-term gains across multiple domains, particularly in cases where language delays persist despite intervention.

Future research should replicate these findings in larger, more diverse samples to verify the generalizability of these results. Moreover, it is crucial to explore how these findings align with international literature on developmental cascades and intellectual disability, broadening the potential impact of this study beyond the Chinese context. The results offer important clinical and educational implications for designing comprehensive, multidisciplinary interventions aimed at optimizing developmental outcomes for children with ID.

## Data Availability

The raw data supporting the conclusions of this article will be made available by the authors, without undue reservation.
